# Navigating Stigma and Discrimination: Experiences of Migrant Children with Special Needs and Their Families in Accessing Education and Healthcare in Hong Kong

**DOI:** 10.3390/ijerph19105929

**Published:** 2022-05-13

**Authors:** Kim Kwok, Sylvia Kwok Lai Yuk Ching

**Affiliations:** Department of Social and Behavioral Sciences, City University of Hong Kong, Hong Kong, China; scyckwok@cityu.edu.hk

**Keywords:** migrant children, disability rights, special needs, stigma, education access, healthcare access

## Abstract

This paper explores the barriers to supporting South Asian (Pakistani, Nepalese and Indian) migrant children with special needs and their families encountered navigating Hong Kong’s special needs system and accessing education and healthcare services. It adopts concepts of stigma and disability rights. It draws on semi-structured interviews with fifteen South Asian children and young people with special needs (age 5–21; M = 10; F = 5) and their families, and seven professional practitioners based on the qualitative descriptive method. Informants experienced intersecting stigmatization that included (1) institutional exclusion, (2) daily life microaggressions, and (3) misunderstandings and a lack of awareness education. Simultaneously, some were empowered through (4) support and accommodation, and (5) spiritual support from religion. This paper reveals a paradox embodied by Hong Kong; it is an Asian multicultural city committed to embracing the vision of disability inclusion while failing to ensure necessary support to reduce the stigma experienced by culturally diverse children with a disability. It makes recommendations based on a socioecological framework and concludes that concerted efforts by relevant authorities and organizations should be made to reduce stigma by taking into consideration the intersecting stigmas, specific resources unique to migrant communities, disability rights and cultural sensitivity.

## 1. Introduction

Stigma is a significant social determinant of health and a driver of poor health outcomes [1,2,3]. Stigma can be manifested in negative stereotypes that cause discrimination and disadvantages for stigmatized persons [4,5]. However, most health-related stigma studies concentrate on an individual level of stigma experience, limiting a broader socioecological perspective to understanding the stigmatization process and interventions [3,5,6]. Increasing international scholarship has raised concern about the potential risk to culturally diverse children’s disability rights [7,8,9,10], a group that currently makes up a sizeable proportion of Hong Kong’s young people [11]. Despite indicators of rights deprivation and lobbying in Hong Kong [12,13,14], attention to children and youth with special needs has not been extended to culturally diverse groups who remain under-represented in research studies, suggesting a gap in the literature and a failure to recognize culturally diverse children’s disability rights, hindering the development of a truly inclusive society.

Hong Kong is an Asian multicultural city committed to the United Nations Convention on the Rights of Persons with Disabilities (hereafter UNCRPD) and formally embracing the vision of a disability-inclusive society [15]. Scholars argue that disability rights can be exercised only through institutional mechanisms inside and outside the government [16,17,18]. Culturally diverse populations (including migrants, refugees and ethnic minorities) with disabilities may encounter more difficulties accessing appropriate education and healthcare services resulting from complex barriers such as language use [7], discrimination and stigma, and lack of culturally sensitive information and intervention [8,19]. A multicultural society with culturally diverse people with disabilities but insufficient institutional support to accommodate their specific needs may challenge the authorities’ stated goal of disability inclusion.

This paper explores the barriers to support that South Asian migrant children with special needs and their families encountered navigating Hong Kong’s special needs system and accessing education and healthcare support services to fill the gap in the literature and policy attention. Conceptually, the paper departs from a socioecological conceptualization of stigma and disability rights perspective. It contributes to scholarship on migrant children with a disability by advancing our knowledge base through examining the paradox embodied by Hong Kong, i.e., it embraces disability inclusion while failing to ensure necessary support to reduce the intersecting stigma experienced by culturally diverse children with a disability.

## 2. Literature Review

### 2.1. Stigma, Discrimination and Mental Well-Being

Stigma has been generally recognized as a social determinant of health, and a major driver of poor health [1,2,3]. According to Goffman, stigma occurs when an “undesirable difference damages the individual’s reputation and …. [disqualifies them] from full social acceptance” [20] (preface). In the case of disability and disease, neither impairment nor disease stigmatizes someone, but other people in society [1]. Most health-related stigma studies concentrate on the individual level of stigma experience, limiting a broader understanding of stigma and interventions [3]. Stangl and colleagues [3] propose a framework of health stigma and discrimination which incorporates the multi-level socioecological conceptualization of the stigmatization process and includes three main dimensions: factors, manifestation and outcomes of stigma. Regarding factors or sources of stigma, scholars have identified institutions, the general public and the self [5,6]. Among institutions, policy makers, health care providers, and public health officials can channel and intensify stigma deriving from fear and blame against those marginalized in society [21,22]. Self-stigma arises from the internalization of stigma by stigmatized individuals and their associates, usually caregivers/parents. Stigma and self-stigma can act as barriers to caregivers’/parents’ coping with their children. Parents of children with a disability may experience stress and internalize stigma, which may unfavorably affect their emotional circumstances, problem recognition and help-seeking decisions [6].

When stigma is applied to people, it is usually manifested in stereotypes based on misconceptions, such as believing that people with mental illness are incapable of holding down a “real” job or living independently [4]. These negative stereotypes may cause discrimination contributing to unequal outcomes for the stigmatized person [5]. In daily life, stigma and discrimination can be manifested in the form of microaggressions, subtle verbal or behavioral insults, conveying “a hostile or derogatory message” [23]. Children and youth with disabilities or from diverse cultural backgrounds experience bullying, an example of microaggression, more frequently because of their minority and disapproved social status [24,25].

Negative experiences of stigmatization have devastating outcomes on the mental health and quality of life of stigmatized individuals and their families, such as a sense of shame, anxiety, and depression [26,27], lower self-esteem and self-efficacy [26], reduced life satisfaction and social adaption [28] and social isolation [29]. Stigma also acts as a barrier to uptaking health information, accessing services and help-seeking [2]. In the public domain, it can result in reduced acceptance, rejection and social exclusion [30], leading to deprivation of rights and equal opportunities for people with disabilities in various aspects of life. Stigma can also have effects on institutions and organizations, including policies and the quality of health services [3].

### 2.2. Intersecting Stigma

Intersecting stigmas occur when people are marked with multiple stigmas that are based not only on their disability but also in combination with other social statuses, such as gender, sexual orientation, race, economic circumstances and cultural background [31,32,33]. As multiple stigmas are often correlated and interrelated, experiences of simultaneous membership of multiple stigmatized groups can generate severe and extensive vulnerabilities and risks [3,33]. For example, Latina women with mental illness in an urban ethnic minority community experienced a multi-layered stigma that was not merely mental illness-related but also based on their ethnicity, gender and poverty. These interlocking effects could increase HIV risk and a sense of disempowerment [31]. Intersecting forms of stigma are a common reality [33]; adopting intersecting stigma in this study provided insights into the interplay of disability with other axes of social status such as ethnicity to impact the severity of disadvantages experienced by migrant children with special needs.

### 2.3. Children’s Disability Rights and Access to Support Services

Historically, people with a disability have been regarded as needing charity and often their rights are discounted by mainstream society [34]. The disability rights approach, promoted by UNCRPD [35], presents a broader understanding of disability and a paradigm shift that goes beyond the traditional medical and social models of disability. It has shifted the perspective from understanding people with disabilities as objects of medical treatment, charity, and social protection to perceiving them as equal members of society, with all accompanying human rights [36], including rights to rehabilitation, reasonable accommodation, health, education, and employment.

According to the United Nations Convention on the Rights of the Child (UNCRC), children with a disability “should enjoy a full and decent life” and states should “ensure that the disabled child has effective access to and receives education, training, health care services, rehabilitation services, preparation for employment and recreation opportunities in a manner conducive to the child’s achieving the fullest possible social integration and individual development” [37] (Article 23). Deprivation of children’s disability rights could negatively impact their educational, physical, cognitive, and social development, reinforcing or increasing social inequality and affecting future opportunities with lifelong consequences for the child [16,17]. Scholars have raised concerns about the potential risk to culturally diverse children’s disability rights because of disparities in healthcare utilization [10,38] and the under-representation of minority students in special education [9]. Suggested explanations for the additional difficulties they face accessing support services and education include language barriers [7], stigma and discrimination [8,19], different understandings of disability [7], difficulty in navigating challenging healthcare systems [10] and lack of culturally sensitive information and intervention [8].

The exercise of disability rights requires governments to uphold the principles of equality, prevent discrimination of rights based on disability [16,17], and provide institutional support inside and outside the government, including law reforms and policy changes in industry, education and empowerment of individuals and communities to advocate for disability rights [34,36]. Examples of institutional support external to the government include the enhancement of community-based support services and disability rights advocacy groups [39,40]. It is argued that without institutionalized mechanisms, disability rights will not be automatically exercised or mainstreamed because governments have additional priorities and people are not informed of their rights [17,18].

China is a signatory to both the UNCRPD and the UNCRC. The Hong Kong government’s vision of a disability-inclusive society embraces the adoption of the UNCRPD principles in devising rehabilitation policies and plans [15]. However, insufficient institutional support to achieve disability rights for culturally diverse minority children with a disability may undermine the achievement of a disability-inclusive society.

The concept of disability rights, together with the framework of the stigmatization process based on Stangl and colleagues [3], will be employed as a lens not merely for interpreting and discussing the data, but also for making recommendations for practice.

## 3. South Asian Migrant Children in the Special Needs Context in Hong Kong

The migration of South Asian people to Hong Kong has a long history dating to the beginning of British colonization in the 19th century. In 2016, South Asian people (primarily of Indian, Pakistani and Nepalese origin) constituted the third largest group of ethnic minorities in Hong Kong, numbering 84,680 [11]. Although some South Asians are affluent and enjoy a high social status, most experience economic deprivation, daily life discrimination and social exclusion [41,42]. Despite their long-standing residence, South Asian people are perceived as “racialized outsiders”, an economic burden and culturally and morally inferior to mainstream Hong Kong Chinese, ideas probably related to deep-seated unspoken racism in Hong Kong [43].

Existing public and scholarly attention on South Asian children and youth has largely focused on their language acquisition, education experiences and employment difficulties [44,45,46]. Language issues pose challenges for all South Asian students. However, these are the most serious for South Asian students with special needs because they are usually channeled into inappropriate learning environments resulting from critical shortages of places in English Medium of Instruction (EMI) schools and EMI special schools [13,14,47]. In the 2013–2014 school year, the dropout rate of ethnic minority students with special needs in all subsidized non-special schools was 57%, much higher than the average 5% for all students with special education needs [13], suggesting that many ethnic minority students found the challenges of a Chinese learning environment too great. Moreover, general stereotypes in wider society, such as perceptions that South Asian students have a low motivation to learn and their parents are uninterested [48], amplified South Asian students’ learning difficulties.

While discrimination and social stigma persist [49,50,51], general understanding and awareness of people with a disability and their rights to equal opportunity have increased in the wider Hong Kong society [41]. This is most probably the result of increased resource allocation to reinforce UNCRPD values and promote a disability-inclusive culture [15]. Subsidized assessment and support services are also fairly comprehensive [52]. Nevertheless, despite policy lobbying for increased support [12,13,14], few measures have been implemented to accommodate the specific circumstances of ethnic minority children with special needs except for the newly established Parent Resource Centres for ethnic minorities [15]. Moreover, it is reported that ethnic minority populations are disengaged from healthcare services and information, mainly due to communication barriers and inadequate translation services in medical settings [47,53]. In view of the above indicators of policy and service delivery gaps and the underrepresentation of culturally diverse minority children with special needs in research studies in Hong Kong, this study aims to fill the gaps and highlight the disability rights of this specific group of children.

## 4. Materials and Methods

### 4.1. Qualitative Descriptive Approach

This paper draws on interviews with fifteen South Asian children and young people with special needs and their families in Hong Kong to explore their experiences of navigating the special needs education and healthcare system. Qualitative description ([54] p. 867, [55]) was applied because it is “ideal for effectively identifying observations and constructs emerging from narrative-based data” and does not aim to apply “a specifically defined lens or theoretical approach to data interpretation” [54] (p. 867).

### 4.2. Data Collection and Informants

Ethical approval was obtained from the first author’s former institute, Caritas Institute of Higher Education. The research team, including one research assistant of Pakistani origin, established contacts with NGOs and schools through which informants were referred. In line with the qualitative description approach, families from the Pakistani, Nepali and Indian communities were recruited using purposive sampling, data reaching saturation with a sample size of fifteen [56]. The inclusion criteria were: (1) self-identifying as belonging to one of the South Asian ethnicities; (2) having at least one child assessed as having special needs or waiting to be assessed. The study adopted the Hong Kong government’s definition of special needs as children and students whose learning difficulties make it more difficult to learn than other children [57]. The definition encompasses nine categories, the principal ones being children with intellectual disability (ID), autism spectrum disorders (ASD), attention deficit/hyperactivity disorder (AD/HD), hearing impairment (HI), and speech and language impairments (SLI). The authors prepared a semi-structured interview guide to include support and barriers experienced in navigating education and healthcare services, interactions with service professionals and co-ethnics in migrant communities, and specific issues (e.g., religion) relevant to South Asian families (see Appendix A).

Six families were of Pakistani origin, five Nepali, and four Indian (see Table 1). The ages of the children ranged from 5 to 21 years. All were born in Hong Kong and some lived for a couple of years during their early childhood in their parents’ country of origin. In-person interviews were held on an individual or family basis. In most interviews, the researchers were able to talk to at least one child and one parent; sometimes a sibling or other relative participated. All main informants (parents of children under 18 and one adult informant) provided informed consent before the interview and were informed of their rights to withdraw from the interview without any consequence. Interviews were conducted using a collaborative approach and open-ended questions such as “Please share your experiences about reaching out for services and suitable schools”. Where informants seemed uncomfortable about responding to open-ended questions, semi-structured questions were used to minimize their unease. Urdu-English and Nepali-English community interpreters were used in some interviews to facilitate better communication and rapport. All interviews except one were audio-recorded and transcribed and observations were documented in the form of field notes. All children except one underwent assessment; eight were diagnosed as having weak/borderline intelligence functioning (intellectual disability), five had autism spectrum disorder (ASD), four had speech delay, two had attention deficit and one had hearing impairment. Pseudonyms are used throughout this paper to ensure informants’ anonymity and confidentiality.

In addition, the research team interviewed seven professionals, two teachers from schools with a concentration of South Asian migrant students, four social workers who worked either in a Parent Resource Center or a family service unit serving ethnic minority parents, and one ethnic minority worker. The main selection criterion was that informants had experience working with South Asian families and children with special needs for at least six months. The interviews covered their experiences and perceptions of the struggles faced by South Asian children with special needs and their families and resources. At the end of the research project, the research team provided two information sessions on general knowledge about children with special needs and services in the Pakistani and Nepalese communities.

### 4.3. Data Analysis

The researchers used qualitative thematic analysis, described as “a method for identifying, analyzing and reporting patterns (themes) with-in data” [58] (p. 78). In the initial stage, the researchers read the interview transcripts and fieldnotes to generate preliminary ideas about the informants’ experiences. The next two stages involved discussing the coding outline/framework and inputting the coding framework into the NVivo software. Based on the coding frame, the data were coded and analyzed by identifying and extracting themes in the transcripts related to the informants’ experiences in accessing education and healthcare and their reactions, community resources and constraints. The extracted themes were then reviewed with the coded data. The initial themes were reviewed several times until the researchers agreed on the final themes [58,59]. The methods to ensure the rigor of this research were: (1) checking ‘summaries of the transcripts and main points with some informants to guarantee the data collected reflected their experiences; (2) prolonged engagement of the first author in South Asian communities through ethnographic participation in mutual help programs and providing fieldwork supervision for social work students working in these communities [60].

## 5. Results

Our findings reveal that South Asian migrant children with special needs and their families experienced intersecting stigmatization consisting of (1) institutional exclusion, (2) daily life microaggressions, (3) misunderstanding and lack of awareness education. Simultaneously, some were empowered through (4) support and accommodation, and (5) spiritual support from religion (see Appendix B).

### 5.1. Institutional Exclusion: “Forgotten Kids” in the Special Needs System

One major concern was that the needs and difficulties faced by non-Chinese-speaking children and families were neglected.

In Hong Kong, all subsidized (government or aided) special schools for students with moderate and severe disabilities operate with Chinese Medium Instruction (CMI). The only EMI special school is privately-run with high tuition fees. The father of a 13-year-old Indian boy, Sunny, who attended a CMI special school, complained:

*English school is a big problem here in Hong Kong … English schools are only available to wealthy people … They expected too much, they asked for around HK$12,000 per month … They also expected that we should come from somewhere with a car and from the high-class residence …tiring looking around, you know… So we are forced to put our kid in a Chinese-speaking school now … Maybe they [authorities] think we don’t care about it [education] … children like my son are forgotten kids.*

Jaanya (girl, 11, ASD, Nepalese) also attended a CMI special school. Her mother attributed Jaanya’s lack of progress to a language issue: “*She just goes to school for a whole day … she is not learning much, just going for passing the time … there might be some language barriers … all in Chinese*”.

Outside special schools, subsidized training, such as speech therapy and occupational therapy, was mostly provided in CMI through mainstream schools, hospitals or rehabilitation service units. The parents of Jasir (boy, 8, ASD, Indian) had to work harder to financially support him to attend English private training to avoid missing a critical phase of his development: “*We have no option … unfortunately we have to go to the expensive [private] one [for training in English]*”.

Language issues were also identified in medical settings. Although translation services were provided [47], they did not benefit our informants because they were not informed about them. The mother of Milan (boy, 15, ID, Nepalese) explained: “*I did not know about the interpreter, where can I find it? … A friend helped me [interpreting], but difficult to understand …sometimes I did not go*”.

The absence of subsidized EMI special school places, inadequate provision of EMI screening and special needs training, and insufficient promotion of medical interpretation services are examples of institutional disregard. This can be understood as structural discrimination occurring not necessarily in a way that one person discriminates against another but resulting from “accumulated institutional practices that work to disadvantage” some people even “in the absence of individual prejudice or discrimination” [5] (p. 372). One such disadvantage is that this neglect left less affluent South Asian children with special needs with no option but to enter a Chinese learning environment that might not be conducive to their age-appropriate development [61,62].

### 5.2. Daily Life Microaggression and Reactions

In the context of daily life, informants reported negative experiences in the form of microaggressions such as discrimination, bullying and blaming. At the same time, we identified some emotional and behavioral reactions like embarrassment, self-blame and social isolation that might arise in response to these negative experiences.

Naaz (boy, 21, ID, Pakistani) was once hospitalized because he became aggressive in the special school he attended. Before his discharge, his mother reported that the hospital doctor told her she should leave Hong Kong: “*You better go back to Pakistan, go back to your hometown, take him away*”. This derogatory message suggested that Naaz, a Pakistani boy with a disability, should not enjoy medical treatment in Hong Kong, disregarding the fact that Naaz and his family were Hong Kong citizens and had a right to receive medical care. Rejecting them in the healthcare system reflects discrimination grounded not merely on disability but also on country of origin and ethnicity.

Microaggression occurred no less within the co-ethnic migrant communities or even family circles. Naaz was bullied by other children in a mosque because of his “strange” pronunciation and behaviors. Naaz recalled the experience: “*They [the children] were aggressive … They laughed at me*”. Relatives accepted him when he was small, but as he became a young man and continued to behave atypically, they reacted with unfriendly comments: “*[They] did not expect he has become like this*”. Naaz’s mother and brother mentioned their “embarrassment” in taking him with them to relatives, resulting in them distancing themselves from their co-ethnic social circles. Naaz’s mother said: “*If I do not understand something? … I go to nobody. I simply do not do it*”.

Jaanya’s mother faced subtle blame from co-ethnic acquaintances through questions like: “*How did that happen?*” and “*Did you eat something wrong?*”. At first, these questions appeared as warm concern, yet implied a subtle accusation based on stereotypes blaming mothers for their children’s atypical development because they must have “done something wrong” during pregnancy or the caring process [63]. Jaanya’s mother showed her sadness, self-doubt and self-blame: “*Uncomfortable … maybe I should have been more careful during pregnancy?*”.

The parents of Maisha (girl, 14, mild ID, Pakistani) were vague or concealed her special needs status during the interview although her diagnosis was clear to the research team when they showed us the medical report. Maisha’s father presented the issue as purely a language one: “*My daughter is having difficulties in written English and Chinese … no other service [training] is needed*”. The family worried that Maisha’s academic results might not fulfill the requirement for moving on to their desired secondary school. When asked if they would try other schools, Maisha replied, “*I am not clever … probably no school will take me … no trying*”. Maisha’s father said the family would arrange Maisha’s marriage if no school would accept her. Here, vagueness about Maisha’s diagnostic status, self-blame and self-devalue was reflected by her “no try” response [64]; these might result from a combination of Maisha’s negative experiences encountered outside her home and her parents’ attitudes, indicated by their disapproval of Maisha’s worth and rights to receive secondary education.

Daily life microaggressions reinforced stereotypes that added to our informants’ accumulated frustration and distress. Parents and some children showed signs of internalizing the negative stereotypes and reacted with self-blame, self-devaluation and problem concealment, which appeared to be linked to their sharing and help-seeking difficulties.

### 5.3. Lack of Knowledge and Awareness Education in the Community

Awareness education was absent while misunderstanding and stigma surrounding special needs issues were identified in the South Asian communities.

Facing their child’s suspected developmental issues, some better-educated parents sought relevant information on the internet. However, others did not know where to obtain useful information and used a wait-and-see approach while experiencing doubt and confusion. It was not uncommon for parents to send pre-school children to their country of origin to live or see a doctor, hoping that the monolingual environment would help their child’s development. Some sent their child to kindergarten in Hong Kong, but communication with kindergarten teachers was poor or not followed up even when parents were alert to their child’s possible developmental delay. The elder sister of Yafir (boy, 11, borderline intelligence, Pakistani):

*My mom remembered the teacher mentioned that … maybe she could not pick up … because of language barrier …there was even a psychologist telling us something … Mom has this concept, she feels that with time he can improve and catch up …He is the fourth son, all other three of us were a bit late at the beginning, many of us too [in the community], but were later OK.*

As in Yafir’s case, the wait-and-see approach resulted in missing pre-school assessment and training, considered a very critical intervention period [61,62].

While some children were diagnosed with autism spectrum disorders or intellectual disability, parents tended to share information about speech delays only in interviews, as in the case of Maisha. All these reflect a common belief in the South Asian communities that their child’s atypical development, especially the “seeming speech delay”, resulted from their minority status living in a multilingual environment (Cantonese, English and their native language), implying that with time and with a better linguistic environment their child could overcome slower development. Besides, South Asian communities have limited understanding of terms such as autism and intellectual disability which remain imbued with notions of stigma and shame [65,66]. Mentioning speech delay only enabled the level of “disability” to be minimized. Therefore, special needs issues were often presented as post-migratory adaptation issues and not handled with sufficient attention or timely intervention.

One social worker observed the absence of timely community education:

*In comparison to local Chinese parents in Hong Kong, I feel that there have been more awareness campaigns in the past 5–10 years, more information on how to train children like those with autism, how to detect autistic behaviors… but we do not have a similar campaign for ethnic minorities.*

### 5.4. Accommodation and Support

Support and accommodation in the family and from relevant institutional professionals played an essential role in facilitating access to information, education and appropriate services.

For example, Aisha (mother of Ifaan, boy, 5, Pakistani) received significant support, including advice and her sister-in-law accompanying her to professional visits. Within four months, they obtained the full assessment for Ifaan in a Child Assessment Centre. Aisha revealed that this would have been impossible “*without the assistance of her sister-in-law*” and other members of her extended family and support from the social worker:

*The worker from SWD [Social Welfare Department] coached me step by step carefully and explained everything in detail … when I didn’t understand I called him, he told me all the procedure…and then he checked for me everything, he checked the whole folder, whole document …*

During the research, we referred Yafir’s mother to a nearby Parent Resource Centre that had a trained community worker of Pakistani origin. Yafir’s mother reported that for the first time in many years, she was able to obtain information about her son’s condition in a comfortable way, where she did not have to ask for language help or feel ashamed as if she had done something wrong.

Some CMI mainstream schools also strived to make adjustments for new South Asian immigrant students. Ms. Ko, the coordinator for students with special education needs in a secondary school, said:

*W* *e use English … we employ a speech therapist who uses English in training … Use Cantonese? I think this is not reasonable. These children do not speak proper Cantonese, how can they understand? If we really do it in this way, the whole world will be diagnosed with speech delay.*

### 5.5. Spiritual Support from Religion

Religion is another important source of support. Ifaan’s aunt explained how God guided them to support Ifaan and his parents:

*It is God who decides all these, so we never complain and blame … blame it’s mommy or daddy’s fault … [God says] the right thing is we need to support him, to support our sister … where we could get support we would recommend her to get.*

Sunny’s family belonged to the Indian Muslim community. After he told us he was a “*special gift from God*”, his father added: “*[we] accept him as someone very special … all good or bad come from God, all will be good at the end… because of this, we [including my brothers and sisters] give him even more love … pay effort to look for what suits him*”.

Our findings revealed that families with predominantly strong extended family support, such as Ifaan’s and Sunny’s, emphasized the role of religion as a positive enabler. Some scholars [67] explain this connection as Islamic belief facilitating a deeper connection between family members and the child and replacing initial superstitious beliefs and questioning why they had a child with a disability.

## 6. Discussion, Implications and Recommendations

### 6.1. Theoretical Implications: Intersecting Stigmas and Disability Rights

This study reveals that while South Asian children with special needs and their families shared some common difficulties with their Chinese counterparts in the wider Hong Kong society [49,50,51], they were confronted with more complex barriers that made their pathway harder. These barriers, conceptualized under the socioecological framework of stigma and discrimination suggested by Stangl and colleagues [3], include structural discrimination manifested through institutional disregard of the rights and needs of culturally diverse children in the special needs system (policy and institutional level), daily life microaggression and self stigma (individual and interpersonal levels), misunderstanding and stigma deriving from South Asian migrant communities, and lack of awareness education in the communities (community and organizational levels). These barriers appeared to adversely impact South Asian children’s access to quality special needs education and healthcare services. These findings point to a paradox of this multicultural city, which claims to uphold disability rights on the one hand, while on the other hand, largely fails to make institutional arrangements to allow culturally diverse children with a disability to enjoy them. The findings echo the international literature that disability rights cannot be automatically exercised without institutional mechanisms inside and outside the government [16,17,18], and disability rights are at risk when the specific needs of the migrant population are not addressed and accommodated [10,38]. Moreover, they deepen our understanding of the stigmatization process by shedding light on how multi-level stigmas and barriers occur beyond the individual level, including within the migrant communities.

This study demonstrates the complexity of these barriers because they result not merely from disability-related stigma and discrimination but an interplay with other axes of disempowerment-language, ethnic origin, and community awareness. The institutional neglect of South Asian children’s language needs and medical staff’s verbal refusal to include them in the healthcare system are evident examples of ethnicity-based discrimination, possibly related to deep-seated but unspoken racism in Hong Kong and long-standing stereotypes about South Asian people [43,48]. This is indicative of how disability can be exacerbated by language and ethnicity. It suggests that while disability contributes to disadvantages in our society, its intersection with language, ethnicity and poor community awareness can further aggravate disadvantaged statuses, increasing the disempowerment of some children with special needs by disability stigma. These findings reiterate the argument that intersecting stigma can generate a more severe and broader range of vulnerabilities [3,33,39].

### 6.2. Practical Implications and Recommendations

As our findings suggest, the comparable knowledge base and awareness level have developed more slowly in South Asian communities than in the wider Hong Kong community. This is most likely due to the lack of attention given to the complex barriers South Asian children and families face and the mainstream special needs system’s failure to undertake proactive awareness education targeted at South Asian communities. This study identifies a gap in the literature. Community-based support groups and advocacy groups are absent from South Asian communities, although they are recognized as a crucial protective factor in international studies, facilitating people with a disability and their caregivers to learn from each other, gain moral support [68], express opposition to social stigma, develop more confidence and engagement with life [69], decrease stress, increase health and well-being [70], and advance policy support [71]. These groups are especially crucial for people with a disability from communities where professional resources are insufficient or inadequately channeled [70], such as the South Asian migrant communities in Hong Kong. To the best of our knowledge, existing self-help groups, parent support groups and advocacy groups for people with a disability in Hong Kong have neither included members from the South Asian communities nor addressed their concerns in advocacy [71,72].

In view of these gaps and the above-discussed barriers and stigmas, this paper makes practical recommendations based on the disability rights concept and the socioecological stigma reduction framework proposed by Stangl and colleagues [3], which include individual, interpersonal, community, organizational and policy levels. At the individual and interpersonal levels, rehabilitation service providers should support individual South Asian children with special needs and their families to cope with experienced stigma, overcome internalized stigma and empower them by articulating their rights to reach out to useful information and services. At the community and organizational levels, rehabilitation service providers and migrant organizations should provide community education to enhance the awareness and knowledge of members of the South Asian migrant communities. Based on the concept of disability rights, these enhancement programs should not portray children with special needs as objects of medical treatment, charity and social protection, but as equal members of Hong Kong society entitled to education, healthcare and rehabilitation services [35,37]. These services should be culturally sensitive [9,10], taking into account these families’ migration backgrounds, language needs, specific doubts, beliefs and community resources, such as family networks and religion, as identified by this study. For example, our information sessions in South Asian communities indicate the value of involving religious organizations in the community in addressing and denouncing the belief that a child’s disability is their parents’ fault. Furthermore, relevant organizations in the special needs system (including hospitals, schools and service NGOs) should take the initiative to provide culturally sensitive stigma reduction programs for their staff. At the policy level, the concerted efforts of related authorities (such as the Hospital Authority, the Education Bureau and the Social Welfare Department in the case of Hong Kong) are required to devise institutional inclusive measures. These include, but are not limited to, policy change and financial support to facilitate community-based awareness programs, support groups and advocacy groups, setting up a subsidized EMI special school and increasing the use of EMI in subsidized special needs healthcare services and training.

## 7. Conclusions

This study contributes to scholarship and practice about culturally diverse children with a disability as follows. First, theoretically, it deepens our understanding of intersecting stigma and disability rights by reinforcing the existing literature that intersecting stigma can generate a more severe and broader range of vulnerabilities [3,33,39], and that disability rights cannot be automatically exercised without institutional mechanisms [16,17,18]. In addition, it sheds light on how multi-level stigma and barriers occur beyond the individual level, prompting us to adopt a broader socioecological perspective to understand stigma and conduct interventions [3]. Second, empirically similar studies [8,10,19] of culturally diverse populations’ disability rights primarily document cases in Western countries. This paper advances our knowledge base by revealing a paradox of an Asian multicultural city, which embraces disability rights but largely fails to make institutional arrangements to allow culturally diverse children with a disability to enjoy them. Third, practically, the paper highlights the significance of community-based awareness education, support and advocacy groups and recommends filling the existing gap through improvement actions at various socioecological levels for policy makers and practitioners. As a whole, this paper concludes that a concerted effort by relevant authorities and institutions in policy and practice should be made to reduce stigma and exclusion by taking into consideration intersecting stigma, specific resources unique to the migrant communities, the rights-based concept and cultural sensitivity.

This study has some limitations. Informants were specifically selected through purposive sampling; therefore, they were more likely to be ready to talk and reflect on their experiences than others who were less accessible. However, cultural restrictions might have inhibited informants from conveying their experiences fully. Our sample was largely concentrated on families with lower socioeconomic status. Difficulties recruiting more affluent informants left gaps in understanding the experiences of Hong Kong’s South Asian communities in this area. Future research should incorporate a more heterogeneous sample to include families from a broader range of socioeconomic and ethnic statuses to offer a more comprehensive picture. Moreover, attention should be paid to similarities and differences in experiences encountered by younger and older migrant children with disabilities.

## Figures and Tables

**Table 1:** Participant children’s demographic information.

Child’s Name	Ethnic Origin	Gender	Age	Diagnosis	Education and Training
Ifann	Pakistani	M	5	Borderline cognitive ability, weak motor skills	Kindergarten; waiting for referral for special training
Naaz	Pakistani	M	21	Intellectual disability, speech delay	Finished special school; no more training
Baheela	Pakistani	F	10	Borderline intellectual disability	Primary 4; special training at school
Maisha	Pakistani	F	14	Intellectual disability, speech delay	Primary 6; special arrangement in exam at school
Yafir	Pakistani	M	11	Borderline intelligence, inattention	Primary 5; waiting for the school for referral of special education training
Azban	Pakistani	M	7	Intellectual disability	Special school
Milan	Nepalese	M	15	Intellectual disability	Special school
Kasmita	Nepalese	F	5	Not yet (suspected ASD; waiting for assessment)	Kindergarten
Taral	Nepalese	M	5	Attention deficit, ASD	Kindergarten; speech and occupational therapies from NGOs
Faneel	Nepalese	M	10	Hearing impairment, speech delay	Primary 4; speech therapy
Jaanya	Nepalese	F	11	ASD	Special school
Jasir	Indian	M	8	ASD, borderline intelligence	Primary 2; earlier intensive training including speech therapy
Aheli	Indian	F	6	ASD	Special childcare center
Kabir	Indian	M	7	ASD, speech delay	Special school
Sunny	Indian	M	13	Intellectual disability	Special school

## Data Availability

The data presented in this study can be available on request from the corresponding author. The data are not publicly available due to confidentiality.

## Appendix A

**Interview Protocol**

This research aims to understand the experiences of South Asian (Pakistani, Nepali and Indian) children and families with special needs in Hong Kong. The main target groups are South Asian families with at least one child diagnosed with special needs or waiting to be diagnosed. Your sharing and information will be most valuable for this research. The following interview will consist of some open-ended questions. It is based on your voluntary participation, and you can withdraw at any time during the process without any negative consequence. The interview will only be recorded upon your consent, and you have the right to stop recording at any time during the interview. All the information collected will be kept confidential and is only for academic purpose.

**Diagnosis**

1 Please share when and how you realized that your child has atypical development or special needs.
2 Why did you decide to seek a diagnosis? Was it a difficult decision?
3 How was the diagnosis process?
4 Are language and finance critical issues during the diagnosis?

**Healthcare and education practice**

5 Please share your experiences about reaching out for healthcare services and suitable schools.
6 What do you do usually to take care of your child’s special needs?
7 How is the division of labor at home?
8 How did you learn to do or request the above-described trainings/services?
9 What is the most difficult part for you in taking care of your child?
10 What do you wish for your child or want him/her to be?
11 Has the outbreak of COVID-19 made the situation more difficult?

**Support systems**

12 Do other members of the family understand your child’s special need? Are other members of the family supportive?
13 How do people usually perceive children with xxx (the name of the specific need/disability) in your community?
14 Does your child encounter discrimination or bullying in school because of their special need?
15 Is the school supportive? Is it difficult to ask for accommodation for your child?
16 Is language an issue for you to obtain support (from school, hospital or social worker)?
17 Does religion play a role in supporting you?
18 Do you share your experiences with other parents in similar situation? How?

## Appendix B

**Table A1:** Themes of Findings Mentioned by Informants.

Informant Child’s Name	Ethnic Origin	Institutional Exclusion	Daily Life Microagres-Sion	Misunderstanding and Lack of Awareness Education	Support and Accommodation	Spiritual Support from Religion
Ifann	Pakistani			✓	✓	✓
Naaz	Pakistani	✓	✓	✓		
Baheela	Pakistani		✓	✓		✓
Maisha	Pakistani	✓	✓	✓		
Yafir	Pakistani	✓	✓	✓	✓	
Azban	Pakistani			✓	✓	✓
Milan	Nepalese	✓		✓	✓	
Kasmita	Nepalese		✓		✓	
Taral	Nepalese	✓		✓		
Faneel	Nepalese	✓		✓	✓	
Jaanya	Nepalese	✓	✓	✓		
Jasir	Indian	✓				
Aheli	Indian	✓				
Kabir	Indian	✓	✓	✓		✓
Sunny	Indian	✓	✓		✓	✓
